# Mendacity: The Tendency to Lie or Deceive. A Cautionary Tale in Obesity Research, Stigma, and Headlining

**DOI:** 10.3389/fendo.2020.598713

**Published:** 2021-01-11

**Authors:** Michael D. Kendig, Margaret J. Morris, Katherine Samaras

**Affiliations:** ^1^ Department of Pharmacology, School of Medical Sciences, University of New South Wales, Sydney, NSW, Australia; ^2^ Diabetes and Metabolism, Healthy Ageing Theme, Garvan Institute of Medical Research, Darlinghurst, NSW, Australia; ^3^ Department of Endocrinology, St Vincent’s Hospital, Darlinghurst, NSW, Australia; ^4^ St Vincent’s Clinical School, St Vincent’s Hospital, Darlinghurst, NSW, Australia

**Keywords:** obesity, fat shaming, discrimination, bias, scientific rigor, psychology

## Introduction

The Obesity section of Frontiers in Obesity has nearly clocked up its fourth year, during which contributors have published over 200 peer-reviewed papers in 28 Research Topics (~700,000 reads), all freely available under open access, to disseminate scientific research into this epidemic. As highlighted in our Grand Challenge, an aim of the section was not only to publish high-quality peer-reviewed research, but to champion those afflicted by obesity through promoting understanding of the underlying science (1). People with obesity are at increased risk of multiple adverse physical and mental health outcomes (2) and face heightened risk of severe health complications and death during the current coronavirus pandemic (3), posing unprecedented concerns for their welfare.

## Stigma in Obesity Research

These health risks are compounded by the pervasive stigma and discrimination against people with obesity across interpersonal and professional domains (4). As articulated in a recent Consensus Statement published in Nature Medicine (5), stigma against people with obesity poses harm to quality of life and mental health and may directly or indirectly perpetuate obesity *via* social or behavioral changes (6). For example, a study employing ecological momentary assessment found that people with obesity reported near-daily instances of stigma, which reduced their motivation to engage in healthy behaviors—particularly for participants reporting greater prior exposure to stigma (7).

Stigma in its various forms compounds the effects of other environmental, economic and social barriers (8) that impede access to, and maintenance of, obesity treatments. Weight discrimination and stigma also manifest within primary healthcare settings (9) and have very real effects on mortality and morbidity. A meta-analysis of studies during the H1N1 influenza pandemic found that obesity was associated with a higher risk of death and poorer health outcomes following infection, but that people with obesity were less likely to receive early access to antiviral treatment, with no association after controlling for this relationship (10). Together, this evidence underscores the importance of studying the physiological and behavioral effects of obesity using targeted questions that advance understanding of the disease while minimizing bias and stigma towards people living with obesity.

A recent study (11) provides a cautionary tale for the design and interpretation of behavioral data in research involving people with obesity. Participants were randomly assigned to complete a series of tasks probing risk attitudes before or after being fed a standardized breakfast. The key task involved participants rolling a three-sided die to receive either 0, 3 or 5 Euros in payment. Crucially, the outcome of the die roll was private and unknown to the researchers. Given that the three outcomes are equally likely in the long run, participants’ honesty could be estimated at the group level (but not for any individual) by analyzing the distribution of rolls reported. In addition to the fasting versus fed manipulation, di Sorrentino and colleagues classified their participants by body weight, and found those with obesity were less likely to report a roll earning 0 Euros and significantly more likely to report a roll earning 5 Euros, compared to chance, either before or after breakfast. Participants of normal weight exhibited a similar tendency to overreport the high-payoff roll of the die when fasted. When sated, however, the distribution of reported rolls did not differ significantly from chance for normal-weight participants.

Di Sorrentino et al. (11) provided direct comparisons between participants of normal weight and those with obesity in their supplementary data. Of the 12 tests reported [3 die outcomes x 2 test conditions (hungry vs. sated) x 2 sexes], one significant difference was found: female participants with obesity were significantly less likely to report a 0 Euro outcome than their lean counterparts. Obesity was not associated with over- or under-reporting an outcome for 11 of the 12 comparisons. The “disconnect” between the title of the article (dishonesty is more affected by BMI status than by short-term changes in glucose) and the largely null effects of obesity is puzzling, particularly considering the paper underwent peer review. Additional methodological concerns with the study—such as the primary outcome, power calculations and corrections for multiple comparisons—have been discussed elsewhere (12).

## Discussion

We note that there are selection biases that serve to promote and suppress the publication of positive and negative results, respectively, and which constitute an ongoing challenge for scientific researchers, reviewers and journal editors alike (13). Viewed in this broader context, the issues with this particular article might be seen as the end product of systemic problems in the incentive structure for publishing academic research, where controversial or eye-catching results are effective in generating media interest and citations, yet often without corresponding scrutiny of the study design and analyses. Thus, while this instance has the potential to perpetuate stigma to people with obesity, the scenario is by no means unique to this field of study. At the time of writing, journal editors have alerted readers that concerns have been raised, with action initiated on August 10th, 2020. Nonetheless, messages from scientists can be taken as truth by the lay public, or presented as such by (social) media, often betraying the nuance inherent in the results of scientific research. Thus, article titles which imply that people with obesity manifest particular positive or negative qualities, regardless of whether they are intended to do so, are problematic.

The recently published consensus guidelines for rigor, objectivity and non-judgmental thinking in obesity research (5) provide an instructive guide in the dissemination of obesity research. Adoption of the guidelines is particularly important given that stigma towards people with obesity extends not only to physical and mental health, but to discrimination in learning (educational) and earning (socioeconomic) domains (14).

Research on the effects of obesity on the body and brain is undoubtedly critical to alleviate adverse health effects and improve quality of life for all. At its best, science can excite and inform public debate. We assert that researchers must strive for consistent, clear, and honest language when reporting obesity research to the scientific community. This, in turn, will work towards reducing stigma and discrimination towards people living with overweight and obesity in the broader community.

## Author Contributions

MM and KS conceived the article. MK drafted the manuscript. All three authors revised and edited the manuscript. All authors contributed to the article and approved the submitted version.

## Funding

MK is supported by the National Health and Medical Research Council (Australian Government; APP1126929). MM is employed by the University of New South Wales Sydney; KS is employed by the Garvan Institute of Medical Research and St Vincent’s Hospital Sydney.

## Conflict of Interest

The authors declare that the research was conducted in the absence of any commercial or financial relationships that could be construed as a potential conflict of interest.
